# Incorporating social vulnerability in infectious disease mathematical modelling: a scoping review

**DOI:** 10.1186/s12916-024-03333-y

**Published:** 2024-03-18

**Authors:** Megan Naidoo, Whitney Shephard, Innocensia Kambewe, Nokuthula Mtshali, Sky Cope, Felipe Alves Rubio, Davide Rasella

**Affiliations:** The Barcelona Institute for Global Health, Hospital Clínic, University of Barcelona, C/ del Rosselló, Barcelona, 171, 08036 Spain

**Keywords:** Social vulnerability, Infectious disease, Mathematical modelling, Global health, Social determinants of health, Health equity, Health inequalities, Health disparities

## Abstract

**Background:**

Highlighted by the rise of COVID-19, climate change, and conflict, socially vulnerable populations are least resilient to disaster. In infectious disease management, mathematical models are a commonly used tool. Researchers should include social vulnerability in models to strengthen their utility in reflecting real-world dynamics. We conducted a scoping review to evaluate how researchers have incorporated social vulnerability into infectious disease mathematical models.

**Methods:**

The methodology followed the Joanna Briggs Institute and updated Arksey and O'Malley frameworks, verified by the PRISMA-ScR checklist. PubMed, Clarivate Web of Science, Scopus, EBSCO Africa Wide Information, and Cochrane Library were systematically searched for peer-reviewed published articles. Screening and extracting data were done by two independent researchers.

**Results:**

Of 4075 results, 89 articles were identified. Two-thirds of articles used a compartmental model (*n* = 58, 65.2%), with a quarter using agent-based models (*n* = 24, 27.0%). Overall, routine indicators, namely age and sex, were among the most frequently used measures (*n* = 42, 12.3%; *n* = 22, 6.4%, respectively). Only one measure related to culture and social behaviour (0.3%). For compartmental models, researchers commonly constructed distinct models for each level of a social vulnerability measure and included new parameters or influenced standard parameters in model equations (*n* = 30, 51.7%). For all agent-based models, characteristics were assigned to hosts (*n* = 24, 100.0%), with most models including age, contact behaviour, and/or sex (*n* = 18, 75.0%; *n* = 14, 53.3%; *n* = 10, 41.7%, respectively).

**Conclusions:**

Given the importance of equitable and effective infectious disease management, there is potential to further the field. Our findings demonstrate that social vulnerability is not considered holistically. There is a focus on incorporating routine demographic indicators but important cultural and social behaviours that impact health outcomes are excluded. It is crucial to develop models that foreground social vulnerability to not only design more equitable interventions, but also to develop more effective infectious disease control and elimination strategies. Furthermore, this study revealed the lack of transparency around data sources, inconsistent reporting, lack of collaboration with local experts, and limited studies focused on modelling cultural indicators. These challenges are priorities for future research.

**Supplementary Information:**

The online version contains supplementary material available at 10.1186/s12916-024-03333-y.

## Background

The rising number of global disasters due to COVID-19, climate change, and conflict, commonly known as the “3 Cs,” have demonstrated that our world is more vulnerable than ever [1]. Among the notable global health risks are infectious diseases, which pose a dire threat. Even with medical innovations advancing the science of disease prevention, infectious diseases still accounted for over 13 million deaths globally in 2015 [2]. Global increases in urbanisation, deforestation, trade, and transport make our modern world more vulnerable than ever to the rapid spread of infectious disease [3]. Most recently, the COVID-19 pandemic resulted in an estimated 18 million deaths worldwide in just the first year [4]. Inequality often exacerbates the impact of infectious disease and vice versa [5]. The current global crisis of rising inequality and poverty, coupled with easier circulation of disease due to globalisation, creates an urgent need for social vulnerability to be addressed in infectious disease research and management [6].

Recently coined in the natural hazard and disaster fields, the term social vulnerability (SV) is used to describe limited resilience during crises resulting from societal and social systems [7]. Socially vulnerable populations face challenges in anticipating, responding to, coping with, and recovering from disasters, including disease outbreaks [8]. SV is not simply the biological processes that affect disease dynamics but rather the conditions and environments that confer vulnerability and therefore impact disease progression. The 3 Cs have caused a major setback to global poverty and SV, which evidence shows has been unprecedented in recent decades [1, 9]. With the rise of the 3 Cs, the SV field is growing in momentum.

SV is multidimensional with ongoing research being done to define and measure it [8]. However, various researchers have attempted to define SV. The United States (US) Centers for Disease Control and Prevention (CDC) developed a SV index that incorporates age, poverty, household composition, race, and access to a vehicle, among other factors [7]. In discussion with various experts and research around what social dimensions affect disease progression, the following categories were identified as contributors to the concepts of SV: vulnerable populations, the social determinants of health (SDH), culture, knowledge, attitudes, and practices (KAP), geographic location, and contact and movement behaviour. Examples are outlined below.

Vulnerable populations face disproportionate hardships because of their identity or status due to historical injustice, discrimination, and social exclusion. The COVID-19 pandemic, for example, disproportionately affected ethnic and racially marginalised populations as the disease began its spread throughout countries including the US, Brazil, and South Africa [10–12].

SDH, such as poverty, educational attainment, disability, access to housing, household sanitation, and access to clean water, are key contributing factors to differential health outcomes [13–15]. Moreover, cultural behaviour and traditions often influence health. This was notable during the West African Ebola epidemic of 2014–2016, when burial rites that involved the washing and touching of infected corpses contributed to community spread [16]. Culture underpins KAP, which influences disease risk. For example, a survey of American university students found that a young person’s assertiveness in practising condom use varied by cultural identity [17].

Infectious diseases also have geographic hotspots. For example, health outcomes are often poorer in rural areas given limited access to health care [18]. Furthermore, the movement and contact patterns of people greatly impact transmission. People often have specific contact patterns and behaviours, reflected in contact matrices [19]. The type of contact also impacts disease progression. For instance, sexually transmitted infections (STIs) require intimate contact while airborne infections can be spread through casual contact [20].

Given the impact infectious diseases have on human health, prompt and equitable preparedness and response are vital. Mathematical models are commonly used to predict or simulate the progression of infectious diseases and may incorporate interventions or policy responses [21]. Two common types of mathematical models used in infectious disease research are compartmental models and agent-based models (ABMs). Compartmental models assign homogenous populations to a category or “compartment” based on their disease state, with individuals moving between compartments as their disease state shifts [22]. These models frequently use deterministic ordinary differential equations but can also incorporate stochasticity [22]. ABMs, also known as individual-based models, consist of individual, autonomous agents with assigned characteristics [23]. ABMs allow for stochasticity by sampling the characteristics and behaviours of each agent from random distributions or by treating the characteristics as random variables. These agents interact with each other and their environment [23]. There are other types of models similar to ABMs, including network analysis and cellular automata models. Network analysis focuses on the linkages or relationships between actors (e.g. populations, organisations, or individuals) [24]. At each time step, the actor’s status—disease or social—can change depending on their relationships with those around them. Cellular automata models make use of a grid of cells, with the grid representing the geographic area and the cells representing an individual [25, 26].

Considering the use of mathematical models for infectious disease research, and the close ties between health outcomes and SV, models benefit from the inclusion of SV. But despite the demonstrated impact, many mathematical models do not consider SV measures [27–29]. Disregarding SV can lead to biased and unreliable forecasts and neglecting communities at risk, which can impact the success of policy and programmatic interventions [27]. For example, excluding limited access to life-saving healthcare for relevant sub-populations might lead to an underestimation in the predicted number of deaths. Reporting population-level results might also be misleading for specific sub-populations as their outcomes may differ due to their SV status.

Researchers can incorporate SV measures into their models in various ways. Methods to do so include accurately estimating and parameterizing sub-populations, using the validation and calibration processes to ensure real-world dynamics inform the model, and engaging multidisciplinary stakeholders to evaluate the model’s structure, biases, and interpretation [27, 28]. Furthermore, model populations can be stratified or characteristics assigned to individual agents. Recognising heterogeneities allows for a more holistic view of the population and, therefore, supports more accurate and targeted predictions [29]. While select studies highlight SV in their mathematical models, no one has methodically assessed the ways in which SV has been included in modelling.

In this paper, we conducted a scoping review to evaluate and discuss how researchers have incorporated social vulnerability into mathematical models for infectious diseases. Understanding how these models have been developed is a crucial step towards building upon former innovations, sharing strategies for new research methods, and inspiring future models for disease preparedness and response.

## Methods

### Search strategy and selection criteria

Given the goal was to identify key themes and trends, a scoping review was determined to be the most suitable method as opposed to other types of reviews [30]. Scoping review methodology followed the steps outlined by the Joanna Briggs Institute [31] and Arksey and O'Malley methodological framework [32] and was verified by the Preferred Reporting Items for Systematic reviews and Meta-Analyses extension for Scoping Reviews (PRISMA-ScR) checklist [33]. The Arksey & O’Malley framework [32], updated by Levac, Colquhoun, and O’Brien [34], was used to guide the scoping review process. This framework consists of six stages: (1) identifying the research question, (2) identifying relevant studies, (3) study selection, (4) charting the data, (5) collating, summarising, and reporting results, and (6) optional consultation [32, 34].

The following databases were systematically searched for peer-reviewed published articles on 10 June 2022: PubMed, Clarivate Web of Science, Scopus, EBSCO Africa Wide Information, and Cochrane Library. These sources were selected given their breadth and topical relevance. Moreover, the reference list of every second article included in the final full-text review was scanned for additional articles not identified during the database search. Keywords were identified from PubMed and Cochrane Institute MESH terms. Variants and combinations of search terms relating to infectious diseases, mathematical models, and SV were used. See Appendix 1 for search terms for each database. EndNote X9 reference management software was used to import the references and delete duplicates, and title and abstract screening was conducted using the online systematic literature review tool Rayyan. Screening titles and abstracts, reading full-text sources, and extracting findings were done by at least two independent researchers. Any conflicts were resolved through discussion, with the final decision made by the first author if consensus could not be reached.

Eligibility criteria were primary research publications that utilised mathematical modelling for infectious diseases and explicitly considered SV. A mathematical model was defined as a model that simulates a system over time to represent a mechanistic dynamic [35]. Specifically, these dynamics recognise that a population or an individual’s state at a point in time depends on their previous state. They often also include assumptions about the values or distribution of select model parameters [35]. An infectious disease was defined as an illness caused by a pathogen, such as a bacterium or virus, that is able to spread to a susceptible host through an infected person, animal, or contaminated object [36]. From discussions with experts and literature research, structural and individual indicators that may influence a community’s resilience were identified as social vulnerabilities [7]. This included vulnerable or marginalised populations, SDH measures, cultural traditions, contact and movement behaviours, geographic distribution, and KAP indicators.

No timeframe bound inclusion. Only peer-review articles written in English were included. Commentaries, editorials, randomised controlled trials, books, blog posts, and conference abstracts were excluded. Articles that included interventions or economic analyses but did not explicitly incorporate an SV indicator were also excluded. Furthermore, models were excluded if the entire studied population was a vulnerable or marginalised population but no differences were highlighted within the group because this study’s objective was to understand how researchers incorporated SV into model methods and equations.

### Data analysis

The primary outcomes of interest were the techniques used to include SV into a mathematical model and the variables selected. The extracted data were summarised in a table in Microsoft Excel to facilitate identifying gaps, trends, and variations across the selected articles, and to categorise relevant information. The results were further mapped across sub-groups. Qualitative data were analysed using an inductive thematic analysis. Notably, the risk of bias and quality of the studies were not evaluated, as the objective was to simply map the evidence [30, 33]. However, a form of quality control was to only include peer-reviewed publications.

**Fig. 1:**
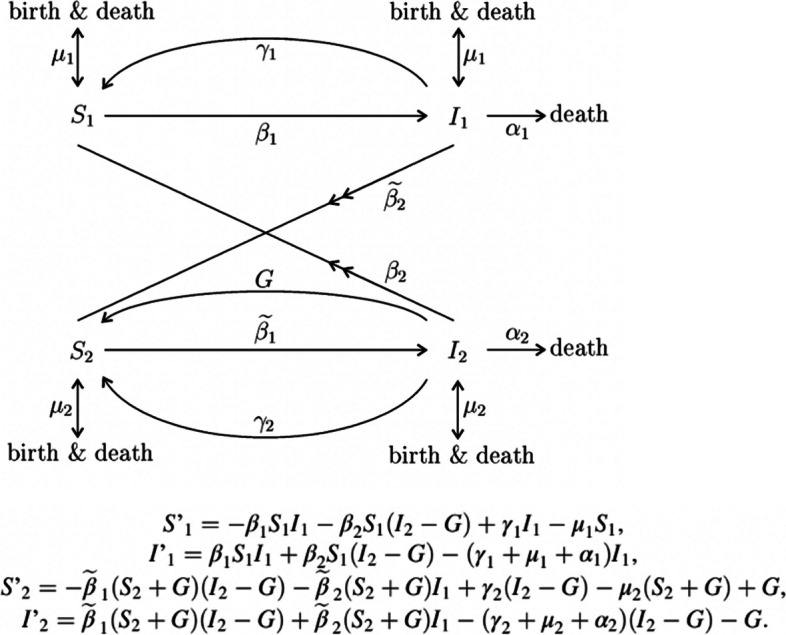
Example of stratifying overall in compartmental modelling: S’_1_ and I’_1_ is a set of equations for the susceptible and infectious housed population, respectively. S’_2_ and I’_2_ is a set of equations for the susceptible and infectious unhoused population, respectively. β_2_ represents interactive transmission between the models Source: Romaszko J, Siemaszko A, Bodzioch M, Buciński A, Doboszyńska A. Active Case Finding Among Homeless People as a Means of Reducing the Incidence of Pulmonary Tuberculosis in General Population. Adv Exp Med Biol. 2016;911:67–76. https://doi.org/10.1007/5584_2016_225. PMID: 26,992,399

**Fig. 2:**
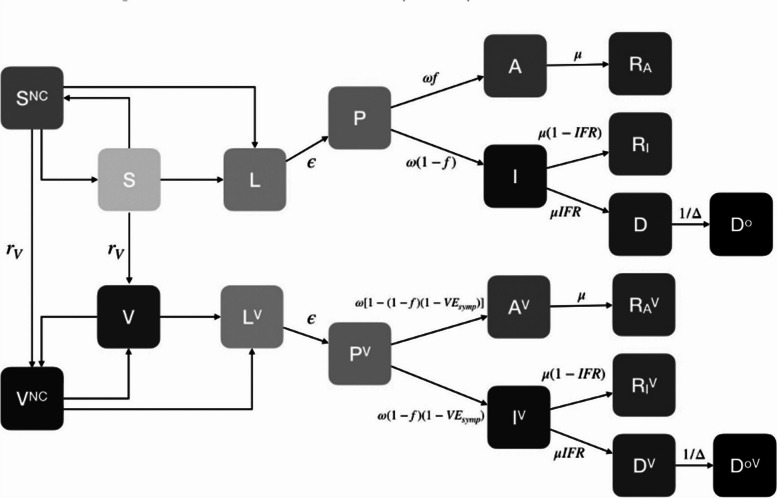
Example of stratifying within in compartmental modelling: Behaviour was modelled in two ways: an increase in the proportion of the population vaccinated led to an increase in non-compliance of preventative measures, and an increase in deaths resulted in an increase in compliance of preventative measures. Susceptible (S) and vaccinated (V) compartments also had susceptible and vaccinated non-compliant compartments (S^NC^, V^NC^, respectively) Source: Source: Gozzi N, Bajardi P, Perra N. The importance of non-pharmaceutical interventions during the COVID-19 vaccine rollout. PloS Comput Biol. 2021: 17(9):e1009346. https://doi.org/10.1371/journal.pcbi.1009346

**Fig. 3:**
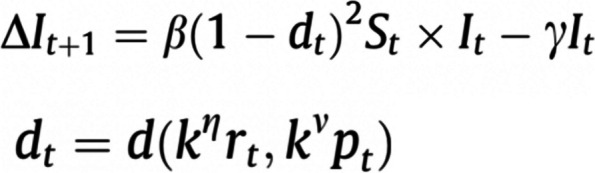
Example of influencing or including parameters in compartmental modelling: The transmission rate β is influenced by *d*_*t*_, a social distancing index between zero and one. *D*_*t*_ comprises measures of civic capital (*k*), the perceived riskiness of the virus (*r*), and policy response adopted over the course of the pandemic (*p*). Included are two civic capital parameters: one based on the internalisation of the externalities (η, belief in other’s well-being) and another on law-abidingness (*v*) Source: Durante R, Guiso L, Gulino G. Asocial capital: Civic culture and social distancing during COVID-19. J Public Econ. 2021 Feb;194:104,342. https://doi.org/10.1016/j.jpubeco.2020.104342. Epub 2021 Jan 4. PMID: 35,702,335; PMCID: PMC9186120

Data collected are listed in Table 1. Regarding the inclusion of SV, indicators were grouped into the following categories: contact and movement behaviour, culture, demographics, geographic location, KAP, SDH, and vulnerable populations. This research categorised the methods of inclusion as *stratification overall*, *stratification within*, *including or influencing parameter(s)*, and *assigning characteristics to agents*. More than one method could be used. *Stratification overall* was defined as the development of separate models for different levels of an SV measure, with possible interactions between these models (i.e. a metapopulation model). See Fig. 1 for an example. *Stratification within* was defined as one model with forking stratification at a point in the model process. See Fig. 2 for an example. *Influencing a parameter(s)* related to adjusting standard or existing model parameters that would be present whether or not SV was incorporated. In comparison, *including a parameter(s)* was defined as the inclusion of a new term related to SV. See Fig. 3 for an example. Although *stratification overall* implies each level of the SV measure has specific parameters, *influencing a parameter(s)* was applicable when the journal article highlighted a change in a particular parameter(s) to reflect an SV dynamic. *Assigning characteristics* were relevant for ABM or network models in which an individual agent or node could have a set of associated variables.

**Table 1:** List of data collected in full-text screening of published articles which included an infectious disease mathematical model with social vulnerability incorporated (*N* = 89)

Metadata	Article details	Inclusion of social vulnerability
Article title	Study setting(s)	Indicator(s)
Date of publication	Disease(s) studied	Indicator category
Author(s)	Mathematical model type(s)	Method(s) of incorporation
Author’s research affiliations	Data sources	If relevant, parameter influenced/ included
Journal	Limitations listed	Stratification method
Article's full reference	Did the model focus on an outbreak?	
	Did the authors conduct a sensitivity analysis?	
	Did the authors calibrat/ fit/ verify the model with real-world data?

The articles were further evaluated to determine if the study focused on an active outbreak (cases exceeding the expected threshold), conducted a sensitivity analysis, and/or calibrated or validated the model. A sensitivity analysis was defined as varying or sampling select parameters to assess their influence on the outcomes of the model [37]. Model calibration is the process whereby parameter values that have limited data are estimated using a model-fitting approach [35].

In order to understand: the extent of collaboration between high-income countries (HICs) and low- and middle-income countries (LMICs), the collaboration with researchers from the study setting, and which countries are leading the research on SV in mathematical modelling, authors’ research affiliations and the study setting were collected. Countries were categorised according to the World Bank regional lists [38].

## Results

**Fig. 4:**
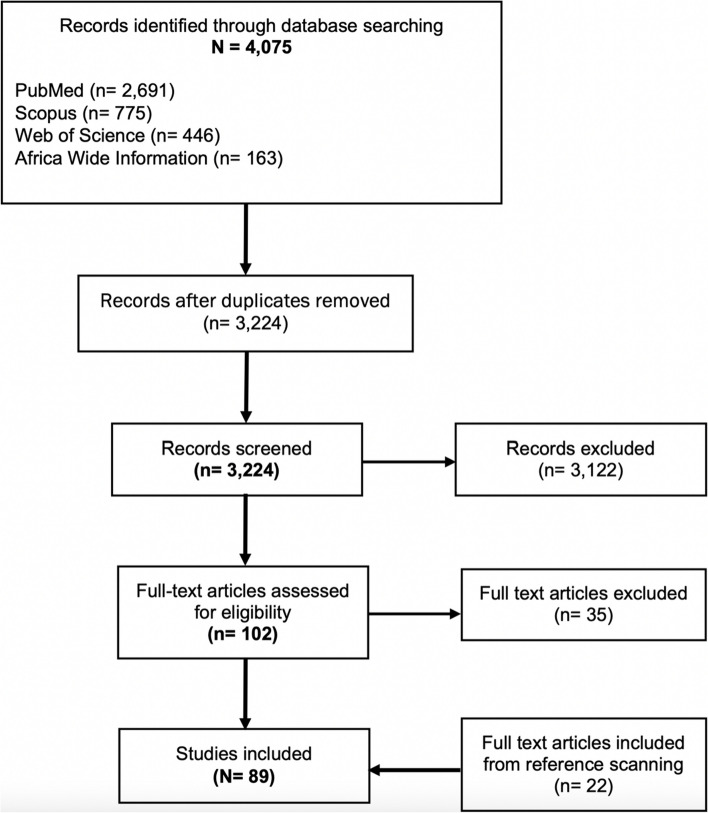
Flow-chart of database searching and article screening process

A total of 4075 studies were identified from the database search: 2691 from PubMed, 775 from Scopus, 446 from Clarivate Web of Science, 163 from EBSCO Africa Wide Information and none from the Cochrane Library. After the duplicates were removed, 3224 articles remained for screening. In total, 3122 (96.8%) were excluded based on the eligibility criteria, leaving 102 articles for full-text screening. Thirty-five of the 102 articles were excluded based on the eligibility criteria. An additional 22 articles were found from full-text reference scanning. The final number of studies included for data extraction was 89. See Fig. 4 and Appendix 2 for the list of articles.

**Fig. 5:**
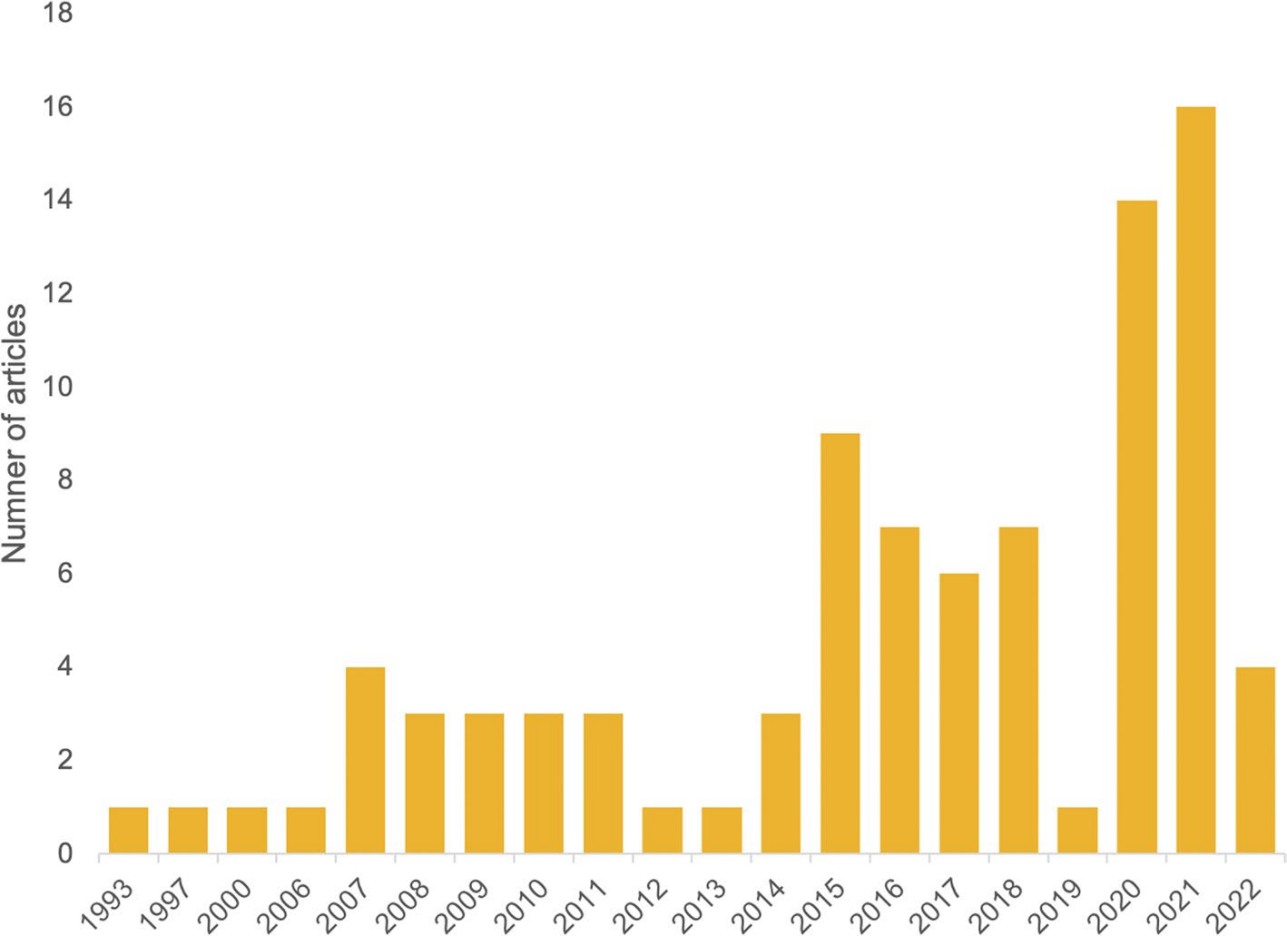
Number of published articles which included an infectious disease mathematical model with social vulnerability incorporated by publication date (to 10 June 2022, *N* = 89)

**Fig. 6:**
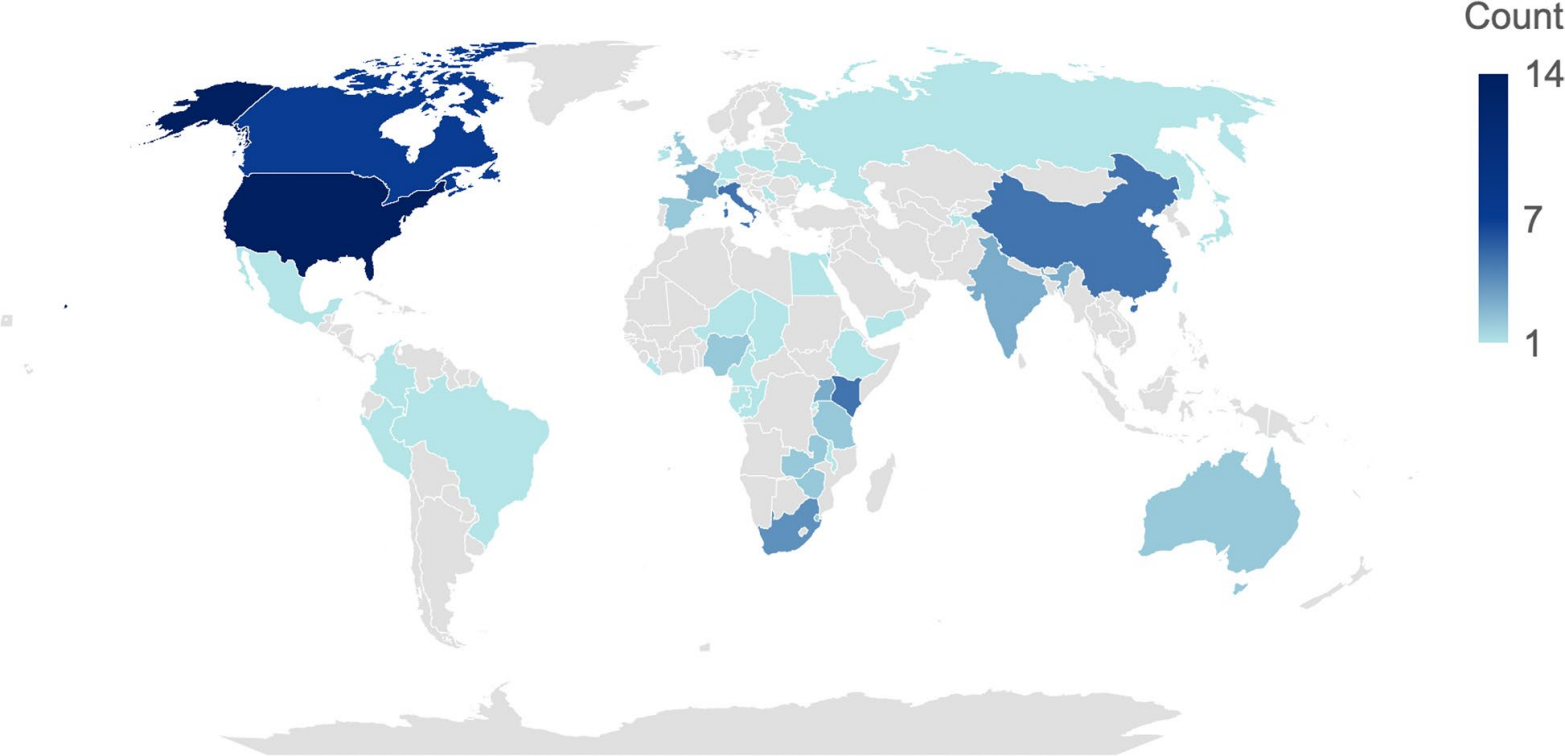
Study setting(s) of published articles which included an infectious disease mathematical model with social vulnerability incorporated (*N* = 89). Twenty-one articles were written about a hypothetical “Setting X” and 12 articles were written about a collection of countries or region(s)

The date of publication ranged from 1993 to 2022 (the year of the database search). Among the studies included, around half (*n* = 42, 47.2%) were published in the last 5 years with a spike in 2020–2021 when 30 studies were published (33.7%). Twenty of those 30 articles focused on COVID-19 (66.7%). See Fig. 5. Geographic locations of study ranged from single settings to several countries or regions. The most common countries studied were the US (*n* = 14, 14.7%) and Canada (*n* = 7, 7.4%), with a dearth in the Middle East and North Africa (*n* = 3, 3.2%) and South Asia (*n* = 3, 3.2%). See Fig. 6. Twelve articles (13.5%) spanned a collection of countries or region(s). Notably, 23.6% (*n* = 21) of all articles modelled a hypothetical “Setting X”. COVID-19 (*n* = 23, 25.0%), influenza (*n* = 19, 20.7%), and HIV/AIDS (*n* = 18, 19.6%) were the most commonly modelled diseases. Strikingly, the fourth most common disease studied was “Disease X” (*n* = 9, 9.8%); a hypothetical disease based on a model of a respiratory, STI, or waterborne-like disease. See Table 2.

**Table 2:** Disease(s) of study in published articles which included an infectious disease mathematical model with social vulnerability incorporated (*N* = 89)

Disease	Count	Percentage
COVID-19	23	25.0%
Influenza	19	20.7%
HIV/AIDS	18	19.6%
Disease X	9	9.8%
Tuberculosis	4	4.3%
Measles	3	3.3%
Cholera	2	2.2%
Malaria	2	2.2%
Respiratory syncytial virus	2	2.2%
Chlamydia	1	1.1%
Cutaneous Leishmaniasis	1	1.1%
Ebola	1	1.1%
Gonorrhoea	1	1.1%
Hepatitis A	1	1.1%
Polio	1	1.1%
Rubella	1	1.1%
SARS	1	1.1%
Schistosomiasis	1	1.1%
Typhoid fever	1	1.1%
Total	92	100.0%

Institutions from HICs generated most of the models (*n* = 56, 62.9%) whereas only five articles were developed solely by LMIC institutions (5.6%). A third of all article affiliations were from the US (*n* = 102, 32.4%). The United Kingdom (UK), Canada, and Italy accounted for nearly another third (*n* = 90, 28.6%). A quarter of articles did not include affiliations from the setting(s) studied (*n* = 18, 25.4%, excluding Setting X). Furthermore, half of articles about LMICs did not include a LMIC author affiliation (*n* = 20, 50.0%). Regarding data source transparency, nearly one in ten studies (*n* = 8, 9.0%) did not list any data sources for parameters in the main text, with the general source (empirical and/or expert opinion) often needing to be inferred from text. A quarter of the articles (*n* = 23, 25.8%) made no clear mention of limitations and six articles (6.7%) made no direct acknowledgement of assumptions.

Among the articles that detailed their limitations (*n* = 66, 74.2%), the following themes emerged: lack of generalizability (*n* = 25, 37.9%); limitations in accounting for disease complexity (*n* = 19, 28.8%); limitations in recognising variability in contact mixing patterns (*n* = 16, 24.2%); limitations in accounting for a social vulnerability (*n* = 15, 22.7%); insufficient data (*n* = 10, 15.2%); assumptions around vaccination (*n* = 5, 7.6%); and limitations in accounting for individual susceptibility (*n* = 4, 6.1%).

**Fig. 7:**
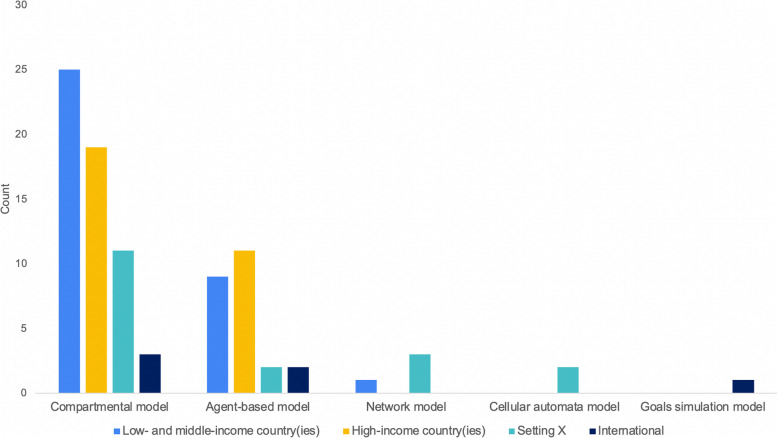
Study setting by mathematical model type in published articles which included an infectious disease mathematical model with social vulnerability incorporated (*N* = 89)

Two-thirds of articles used a variation of the Susceptible, Infectious, Recovered (SIR) compartmental model (*n* = 58, 65.2%), with one in four articles using ABMs (*n* = 24, 27.0%). Four (4.5%) were network models. The remaining three (3.4%) were cellular automata models (*n* = 2, 2.2%) and a goals simulation model (*n* = 1, 1.1%). Among the 58 compartmental models, 62.1% (*n* = 36) included contact or movement behaviour, suggesting metapopulation models were a common means to model SV. ABMs were more frequently associated with HIC institutions (*n* = 18, 75.0%) than LMIC institutions. Only six ABM studies included a LMIC affiliation (25.0%), of which only one ABM was developed solely by LMIC institutions. Nearly half of the articles using ABMs were designed for HIC settings (*n* = 11, 45.8%) whereas 37.5% were designed for LMICs (*n* = 9). See Fig. 7. Conversely, compartmental models were more commonly used for LMIC settings (*n* = 25, 43.1%) than HICs (*n* = 19, 32.8%). See Fig. 7. There was also a closer split between compartmental models generated solely by HIC institutions (*n* = 34, 58.6%) and those that included a LMIC institution(s) (*n* = 24, 41.4%). Setting X most often employed the use of a compartmental model (*n* = 10, 58.8%). See Fig. 7. Modelling for COVID-19 and HIV/AIDS made use of compartmental models 91.3 and 72.2% of the time, respectively (*n* = 21, *n* = 13). Conversely, studies modelling influenza used both ABM (*n* = 10, 52.6%) and compartmental models (*n* = 8, 42.1%), with two-thirds of articles about a Disease X employing ABM and network models (*n* = 6, 66.6%).

The majority of articles focused on an active outbreak scenario (*n* = 65, 73.0%), driven by the high number of articles about COVID-19 (*n* = 23, 35.4%) and outbreak influenza (*n* = 19, 29.2%). Among articles about outbreaks, the majority used a compartmental model (*n* = 43, 66.62%). Half of the articles conducted a sensitivity analysis (*n* = 45, 50.6%) and more than half calibrated or validated their models (*n* = 52, 60.5%, excluding theoretical frameworks).

**Fig. 8:**
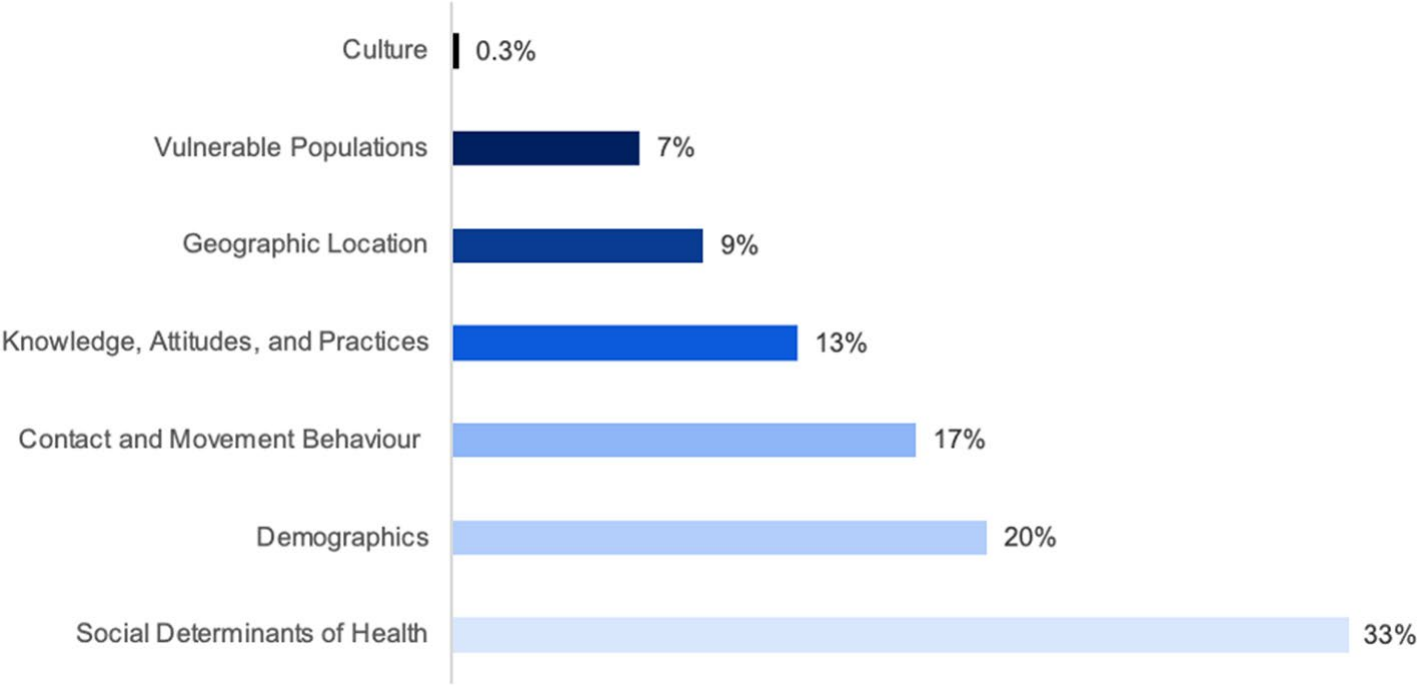
Distribution of the social vulnerability indicators (*n* = 342) by category type in published articles which included an infectious disease mathematical model with social vulnerability incorporated (*N* = 89)

Nearly half of all models included four or more SV indicators (*n* = 42, 47.2%). Among both compartmental models and ABMs, the mean number of indicators included was 3.9. A third of all SV indicators (*N* = 342) were SDH measures (*n* = 114, 33.3%). Demographic factors were the second most commonly used measure of SV (*n* = 68, 19.9%). See Fig. 8. Of individual indicators, the most frequent were contact behaviour (*n* = 47, 13.7%), age (*n* = 42, 12.3%), and sex (*n* = 22, 6.4%; no article considered non-binary sex or other gender identities). The only identified cultural indicator was civic capital (*n*= 1, 0.3%); the concern around others’ well-being and law-abidingness [39]. The most common vulnerable populations studied (*n* = 24) were those living in poverty (*n* = 5, 20.8%), female sex workers (*n* = 4, 16.7%), and men who have sex with men (*n* = 4, 16.7%). Most geographic distribution indicators were based on administrative boundaries such as region (*n* = 6, 18.8%), neighbourhood (*n* = 5, 15.6%), or county (*n* = 4, 12.5%). Unlike other SV categories which were more evenly spread across diseases, among KAP measures and vulnerable populations, more than half were used in studies on HIV/AIDS (*n* = 24, 54.5%; *n* = 14, 58.3%, respectively). See Table 3.

**Table 3:** Indicators (*N* = 342) used in published articles which included an infectious disease mathematical model with social vulnerability incorporated

**COVID-19**	**81**	**100.0%**
*Social determinants of health*	*31*	38.3%
Medical capacity	6	19.4%
Population density	4	12.9%
Access to healthcare	3	9.7%
Health risk	3	9.7%
Access to water	2	6.5%
Employment	2	6.5%
Income	2	6.5%
Poverty	2	6.5%
Sanitation	2	6.5%
Socioeconomic status	2	6.5%
Access to food	1	3.2%
Access to housing	1	3.2%
Household size/ composition	1	3.2%
*Contact and movement behaviour*	*16*	19.8%
Contact	12	75.0%
Movement	4	25.0%
*Demographics*	*15*	18.5%
Age	12	80.0%
Sex	2	13.3%
Race	1	6.7%
*Geographic location*	*9*	11.1%
County	2	22.2%
Region	2	22.2%
Census block	1	11.1%
Cluster	1	11.1%
District	1	11.1%
Neighbourhood	1	11.1%
Province	1	11.1%
*Knowledge, attitudes, and practices*	*7*	8.6%
Adherence to non-pharmaceutical interventions	3	42.9%
Adherence to social distancing	3	42.9%
Knowledge	1	14.3%
*Vulnerable populations*	*2*	2.5%
Nationality	1	50.0%
Poverty	1	50.0%
*Culture*	*1*	1.2%
Civic capital	1	100.0%
**HIV/AIDS**	78	100.0%
*Knowledge, attitudes, and practices*	*24*	30.8%
Condom use	5	20.8%
Number of sexual partners	4	16.7%
Adherence to treatment	2	8.3%
Circumcision	2	8.3%
HIV testing	2	8.3%
Injection sharing and hygiene	2	8.3%
Sexual behaviour	2	8.3%
Information and education campaign	1	4.2%
Knowledge	1	4.2%
Relationship duration	1	4.2%
Treatment use	1	4.2%
Type of sexual act	1	4.2%
*Social determinants of health*	*16*	20.5%
Poverty	3	18.8%
Relationship type	3	18.8%
Choice class	2	12.5%
Access to treatment	1	6.3%
Drug and alcohol abuse	1	6.3%
Education	1	6.3%
Health risk	1	6.3%
HIV status	1	6.3%
Malnutrition	1	6.3%
Marital status	1	6.3%
Medical capacity	1	6.3%
*Demographics*	*15*	19.2%
Sex	10	66.7%
Age	4	26.7%
Race	1	6.7%
*Vulnerable populations*	*11*	14.1%
Men who have sex with men	4	36.4%
Female sex workers	3	27.3%
People who inject drugs	2	18.2%
Poverty	2	18.2%
*Contact and movement behaviour*	*9*	11.5%
Contact	9	100.0%
*Geographic location*	*3*	3.8%
County	1	33.3%
Location	1	33.3%
Urban vs rural	1	33.3%
**Influenza**	76	100.0%
*Social determinants of health*	*26*	34.2%
Household size/ composition	7	26.9%
Health risk	3	11.5%
Employment	2	7.7%
Income	2	7.7%
Population density	2	7.7%
Age gaps between household members	1	3.8%
Age of the head of the household	1	3.8%
Disease risk	1	3.8%
Education enrolment	1	3.8%
Educational system	1	3.8%
Marital status	1	3.8%
Poverty	1	3.8%
Relationship to the head of the household	1	3.8%
SEIFA index^a^	1	3.8%
Social deprivation index	1	3.8%
*Demographics*	*21*	27.6%
Age	15	71.4%
Sex	5	23.8%
Race	1	4.8%
*Contact and movement behaviour*	*17*	22.4%
Contact	13	76.5%
Movement	4	23.5%
* Geographic location*	*8*	10.5%
Neighbourhood	2	25.0%
Poverty	2	25.0%
Region	2	25.0%
Census tract	1	12.5%
County	1	12.5%
*Knowledge, attitudes, and practices*	*3*	3.9%
Adherence to non-pharmaceutical interventions	1	33.3%
Adherence to social distancing	1	33.3%
School attendance	1	33.3%
* Vulnerable populations*	*1*	1.3%
Pregnancy	1	100.0%
**Disease X**	**39**	100.0%
*Social determinants of health*	*17*	43.6%
Employment	3	17.6%
Household size/ composition	2	11.8%
Medical capacity	2	11.8%
Migration	2	11.8%
Socioeconomic status	2	11.8%
Access to healthcare	2	11.8%
Household type	1	5.9%
Income	1	5.9%
Medical hygiene	1	5.9%
Poverty	1	5.9%
*Contact and movement behaviour*	*6*	15.4%
Contact	4	66.7%
Movement	2	33.3%
*Demographics*	*5*	12.8%
Age	2	40.0%
Sex	2	40.0%
Race	1	20.0%
*Knowledge, attitudes, and practices*	*4*	10.3%
Adherence to non-pharmaceutical interventions	2	50.0%
Fear	1	25.0%
Health belief model	1	25.0%
*Vulnerable populations*	*4*	10.3%
Communities in conflict	1	25.0%
Net consumers	1	25.0%
Net producers	1	25.0%
Poverty	1	25.0%
*Geographic location*	*3*	7.7%
Neighbourhood	1	33.3%
Poverty	1	33.3%
Spatial distribution	1	33.3%
**Tuberculosis**	**12**	100.0%
*Social determinants of health*	*4*	33.3%
Population density	2	50.0%
HIV status	1	25.0%
Malnutrition	1	25.0%
*Vulnerable populations*	*3*	25.0%
Unhoused	1	33.3%
Miners	1	33.3%
Poverty	1	33.3%
*Demographics*	*2*	16.7%
Age	1	50.0%
Sex	1	50.0%
*Contact and movement behaviour*	*1*	8.3%
Contact	1	100.0%
*Geographic location*	*1*	8.3%
Neighbourhood	1	100.0%
*Knowledge, attitudes, and practices*	*1*	8.3%
Treatment use	1	100.0%
**Measles**	**9**	100.0%
*Contact and movement behaviour*	*3*	33.3%
Contact	3	100.0%
*Demographics*	*3*	33.3%
Age	2	66.7%
Sex	1	33.3%
*Knowledge, attitudes, and practices*	*2*	22.2%
Adherence to social distancing	1	50.0%
Cooperation	1	50.0%
*Social determinants of health*	*1*	11.1%
Poverty	1	100.0%
**Respiratory syncytial virus**	**7**	100.0%
*Social determinants of health*	*3*	42.9%
Household size/ composition	3	100.0%
*Demographics*	*2*	28.6%
Age	2	100.0%
*Contact and movement behaviour*	*1*	14.3%
Contact	1	100.0%
*Geographic location*	*1*	14.3%
Setting	1	100.0%
**Schistosomiasis**	**7**	100.0%
*Social determinants of health*	*3*	42.9%
Employment	1	33.3%
Household size/ composition	1	33.3%
Sanitation	1	33.3%
*Demographics*	*2*	28.6%
Age	1	50.0%
Sex	1	50.0%
*Geographic location*	*1*	14.3%
Patch	1	100.0%
*Knowledge, attitudes, and practices*	*1*	14.3%
Water contact	1	100.0%
**Hepatitis A**	**6**	100.0%
*Social determinants of health*	*2*	33.3%
Household size/ composition	1	50.0%
Household type	1	50.0%
*Contact and movement behaviour*	*1*	16.7%
Contact	1	100.0%
*Demographics*	*1*	16.7%
Age	1	100.0%
*Geographic location*	*1*	16.7%
Municipality	1	100.0%
*Knowledge, attitudes, and practices*	*1*	16.7%
Food hygiene	1	100.0%
**Malaria**	**6**	100.0%
*Social determinants of health*	*4*	66.7%
Economic conditions	1	25.0%
Medical capacity	1	25.0%
Migration	1	25.0%
Sanitation	1	25.0%
*Contact and movement behaviour*	*1*	16.7%
Movement	1	100.0%
*Geographic location*	*1*	16.7%
Area	1	100.0%
**Cholera**	**4**	100.0%
*Social determinants of health*	*3*	75.0%
Medical capacity	1	33.3%
Malnutrition	1	33.3%
Sanitation	1	33.3%
*Geographic location*	*1*	25.0%
Region	1	100.0%
**Ebola**	**4**	100.0%
*Social determinants of health*	*2*	50.0%
Medical capacity	1	50.0%
Vulnerability	1	50.0%
*Contact and movement behaviour*	*1*	25.0%
Movement	1	100.0%
*Geographic location*	*1*	25.0%
Country	1	100.0%
**Sexually transmitted infection (Chlamydia and Gonorrhoea)**	**4**	100.0%
*Vulnerable populations*	*3*	75.0%
Female sex workers	1	33.3%
People living with HIV	1	33.3%
Youth	1	33.3%
*Contact and movement behaviour*	*1*	25.0%
Contact	1	100.0%
**Rubella**	**3**	100.0%
*Contact and movement behaviour*	*1*	33.3%
Contact	1	100.0%
*Demographics*	*1*	33.3%
Age	1	100.0%
*Social determinants of health*	*1*	33.3%
Disease risk	1	100.0%
**Polio**	**3**	100.0%
*Contact and movement behaviour*	*1*	33.3%
Contact	1	100.0%
*Demographics*	*1*	33.3%
Age	1	100.0%
*Geographic location*	*1*	33.3%
Region	1	100.0%
**Cutaneous Leishmaniasis**	**1**	100.0%
*Geographic location*	*1*	100.0%
Country	1	100.0%
**Severe acute respiratory syndrome**	**1**	100.0%
*Knowledge, attitudes, and practices*	*1*	100.0%
Avoidance behaviour	1	100.0%
**Typhoid fever**	**1**	100.0%
*Social determinants of health*	*1*	100.0%
Socioeconomic status	1	100.0%

^a^SEIFA is a continuous variable and summarises average socio-economic characteristics, including education, occupation, and wealth and can be used to describe the distribution of social and economic well-being

**Fig. 9:**
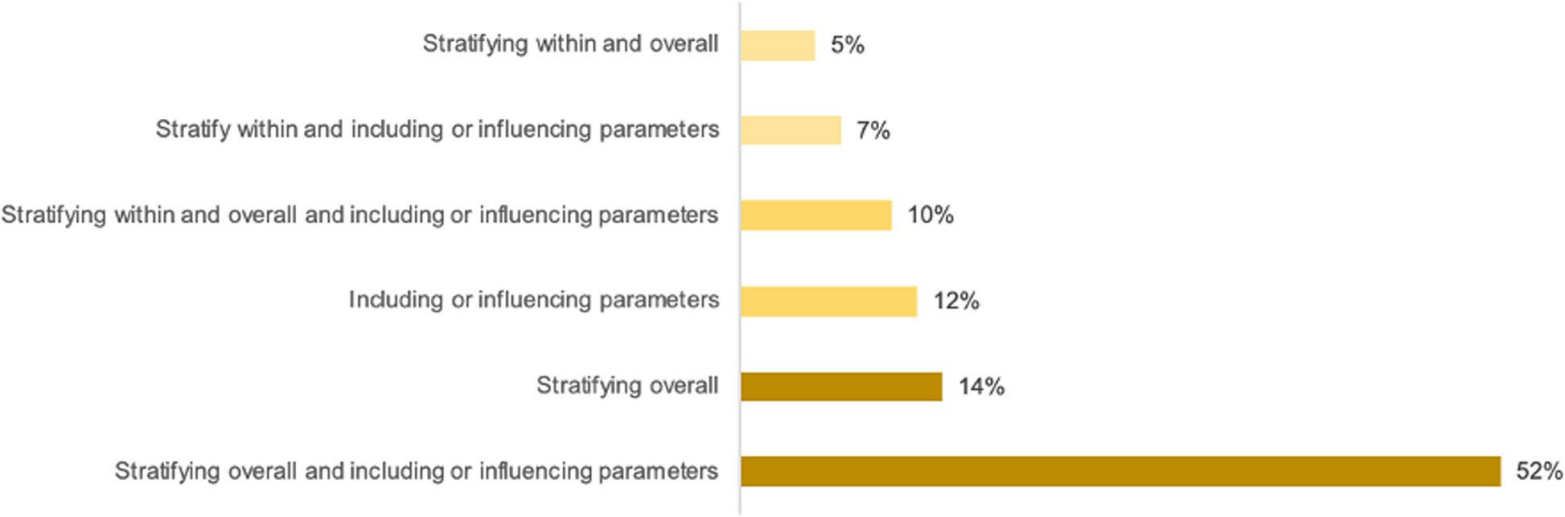
Methods of including social vulnerability indicators in infectious disease compartmental models. See “ Methods” section for an explanation of the approaches

SV was modelled using several methods. Compartmental models utilised a mix of *stratification overall*, *stratification within*, and/or *including or influencing parameter(s)*. As part of their methodology, 44 articles incorporated *stratification overall* (75.9%), 13 articles included *stratification within* (22.4%), and 47 articles involved *including or influencing parameter(s)* (81.0%). The single most common method for compartmental models was to both *stratify overall* and *include or influence parameter(s)* (*n* = 30, 51.7%). See Fig. 9. *Assigning characteristics *was a method exclusive to ABMs and network models. In these cases, SV indicators were assigned to individual agents. Based on their demographic profile, the individual had a probabilistic routine that they followed which influenced their disease state and health outcomes. Additionally, researchers could delineate the virtual space (neighbourhoods, villages, census tracts, etc.) with predefined locations for activity. Agents can have area-associated characteristics and move between areas [23]. Among ABMs, most indicators were SDH measures (*n* = 43, 34.7%) and demographic variables (*n* = 32, 25.8%). Specifically, age, contact behaviour, and sex were the most common individual indicators (*n* = 18, 14.5%; *n* = 14, 11.3%; *n* = 10, 8.1%, respectively). The method of *Other* was only relevant for cellular automata models (*n*= 2), which focuses on spatial distribution using a grid to define a geographic area [25, 26].

Age, poverty, and sex were the most common SV indicators in compartmental models (*n* = 22, 11.2%; *n* = 10, 5.1%; and *n* = 10, 5.1%, respectively). They were most frequently modelled through *stratification overall and including or influencing parameter(s)*. Age was included through a combination of age-structured models and age-based contact rates or matrices (*stratification overall and including or influencing parameter(s)*, *n* = 15, 71.4%). Poverty was also introduced through *stratification overall* (*n* = 6, 60.0%) and adjusting the contact rate (*n* = 4, 40.0%). Additionally, scalars were added to adjust parameters such as transmission, recovery, and mortality rates to account for poverty-related issues like malnutrition, decreased treatment, and overcrowding (*n* = 5, 50.0%). Separate models for males and females were constructed to incorporate sex (*stratification overall*, *n* = 9, 90.0%).

Select SV indicators were modelled using innovative approaches. For example, an ABM for Disease X incorporated the health belief model by basing agents’ health behaviour decisions on perceived susceptibility, severity, benefits, and barriers [40]. In another article, a compartmental model for Setting X included fear [41]. Susceptible agents could be “infected” by fear (through contact with an infected person, by the idea of being infected, or both) which led to protective behaviour or fleeing which increased the spread of Disease X [41]. Civic capital was factored into a compartmental model for COVID-19 in Italy by including key parameters [39]. In calculating the number of infected people, a social distancing index value was introduced [39]. The index included one parameter representing belief in others’ well-being and another on law-abidingness [39]. Given HIV/AIDS transmission is multifactorial, a power dynamic was incorporated in a compartmental model for sub-Saharan Africa, with stratified compartments for choice-enabled or choice-disabled males and females [42]. Choice class was affected by social interactions and indicated if someone was more or less likely to transmit or acquire HIV [42].

## Discussion

The COVID-19 pandemic and rising health emergencies due to climate change and conflict have highlighted the world’s vulnerability to infectious diseases. In response, there has been an urgent call to foreground social vulnerability in infectious disease mathematical modelling [27]. Inclusion is vital for more accurate, bespoke results, implementing effective interventions, and for the integration of social justice into equitable policy-making. This is the first study to thoroughly review the measures and the methodologies used to incorporate social vulnerability into mathematical disease models. Social vulnerability was not considered holistically. Only one article included a cultural and social behaviour indicator, whereas routine demographic indicators, namely contact behaviour, age, and sex, were the most common across model types. For compartmental models, researchers most often constructed distinct models for each level of a social vulnerability measure (e.g. separate models for males and females, with possible interaction between) and varied the parameters. For agent-based models, various characteristics related to social vulnerability were assigned to hosts.

As aforementioned, the concept of SV and the indicators encompassing it have yet to be standardly defined [8]. Methodological challenges, data quality and access gaps, and conceptual challenges limit the development of consistent measures [8]. In this study, SV was mostly measured through age, sex, and contact and movement behaviours. While pertinent, these indicators do not reflect a holistic view of SV. Age and sex are often routine indicators in disease modelling. They affect disease dynamics but their representation as SV indicators needs to be examined. While age was included in the CDC’s SV index (and as a result considered as a SV indicator in this study), it was related to identifying vulnerable populations of people aged 65 or older and 17 or younger [7]. This is compared to an age-based contract matrix which may reflect the epidemiological transmission process but may not highlight a condition or environment that confers vulnerability [19]. Sex is highly relevant to the transmission of STIs [42]. However, if it represents vulnerability is based on how sex is modelled and with what intention. In de Boer et al.’s paper, sex reflected the choices men and women could make and the subsequent transmission and acquisition of HIV [43]. Age and sex cannot simply be checkboxes as a means to include SV in modelling. The intention and consideration of how age and sex confer vulnerability needs to be examined.

While models included four indicators on average, this study found that cultural indicators were ignored. This is likely due to a lack of available data, whereas age and sex are routinely collected. Furthermore, many anthropological measures are challenging to objectively quantify. A review of cultural influences on the transmission and outcomes of the COVID-19 pandemic noted that “the social transmission of infectious diseases means that their spread, and hence their impact on a population, is driven in part by social behaviours which, in turn, are shaped by patterns of culturally shared beliefs in that population” [44]. For example, countries which highly value a culture of individualism (focusing on the individual as an autonomous agent, as opposed to part of a collective) experienced lower adherence to preventive measures such as social distancing and mask wearing, resulting in higher COVID-19 prevalence and mortality [45]. Given the potential impact on disease transmission and outcomes, there should be a greater effort to collect, consider, and incorporate cultural measures into infectious disease modelling.

Among models that integrated SV, compartmental models were the most widely used. As a result, *stratifying overall* and *including new or influencing existing parameters*were the most common approaches for incorporating SV measures. This may be because compartmental models are generally easier to develop than ABMs, and stratifying overall (developing a model for each level of a measure) and varying parameters reveal heterogeneity and highlight vulnerable populations [46].

Nevertheless, compartmental models have both their merits and drawbacks. Compartmental models are computationally inexpensive, replicable, and scalable and can be developed in a relatively short time frame [46]. For these reasons, compartmental models are ideal for outbreaks and resource-limited settings, as was the case for COVID-19 and HIV/AIDS (pandemics that disproportionately impacted LMICs) [11, 12, 47], and is affirmed by this study’s results. However, one criticism of compartmental models is that they simplify real-world dynamics, especially as disease states are represented by homogenous populations [46]. The results from this study demonstrate that SV can be successfully introduced into a compartmental model through stratification and adjusting model parameters, but not to the extent of an ABM, which provides a detailed model of individual characteristics and behaviour. The additional flexibility and complexity of an ABM allows for elaborate scenario building, as seen in its frequent use in studies modelling Disease X. On the other hand, ABMs are computationally expensive and more challenging to scale and replicate [46]. Furthermore, the individual-level data they require is often lacking, particularly data on SV [46]. This reality aligns with this study’s findings that ABMs were used primarily for HIC settings and generated by HIC institutions, which often have more resources and data on SV. Given ABMs are well-suited for incorporating heterogeneity, particularly related to SV, more effort should be made to support ABMs by LMICs.

Beyond the structure of the model, researchers have suggested the following approaches should be taken into consideration when incorporating SV: (1) explicitly acknowledging study limitations and assumptions, (2) using validation and calibration (where appropriate) to strengthen results, and (3) engaging stakeholders and authors with relevant and ideally local knowledge on the study population [27, 28]. These considerations are discussed below.

Researchers must communicate their studies’ limitations and contextualise findings related to SV. Transparency around model limitations and how SV parameters are estimated, especially if using expert opinion as opposed to empirical evidence, are both necessary to critique, reproduce, and expand on established work. However, this study revealed that data sources for parameters and research limitations were not always or clearly stated, in particular for measures and parameters of SV. Consistent reporting is needed.

In this study, only half of articles conducted a sensitivity analysis and little more than half validated or calibrated their models. Although not always appropriate (e.g. if exploring theoretical frameworks), model validation, calibration, and sensitivity analyses can strengthen the robustness of results [46]. This is particularly relevant for SV indicators as these measures are often only proxies. Additionally, while complex, examining whether SV has been successfully incorporated into a model is an important part of model evaluation. To do this, researchers may consider conducting sensitivity analyses to compare estimates, trajectories, uncertainties, and other aspects of the model with and without the incorporation of SV.

A quarter of studies did not include an author from the study setting. This was particularly notable in the case of LMICs. Most articles were generated by institutions in the global north. This is in line with another article's finding that most infectious disease outbreak modelling is being done by researchers in the US and UK [48]. The dearth of articles from the global south is a potential indicator of limited collaboration and resources (e.g. limited data, modelling experience, funding, and institutional support). A recent review of infectious disease models found that international collaborations with less-developed countries were limited [48]. The accuracy of model outcomes highly depends on the quality of input parameter values. Local knowledge of the setting is important for ensuring that input data are reliable and context-appropriate. This is especially important for SV, which is often challenging to represent faithfully in models without knowledge of the local context, socioeconomic dynamics, and available data. Collaboration with local stakeholders should be prioritised.

While SV measures have previously been incorporated into infectious disease mathematical models (although researchers may not use the term), there has been a growing recognition to make the concept of SV a mainstay. A key driver of bringing SV to the forefront has been the COVID-19 pandemic. This influence is reflected in this study, which found a spike of published studies in 2020–2022 and found that COVID-19 was the most frequently modelled disease incorporating SV. This momentum can potentially be leveraged to further innovate methods for including a variety of SV measures in mathematical models.

However, with this momentum, care needs to be taken in how SV is incorporated. Not all indicators should be included into a model to avoid overfitting [49]. For calibration, it is necessary to prioritise the most relevant indicators [49]. Furthermore, SV must be integrated thoughtfully and purposefully, and the method of inclusion needs to be appropriate for the disease. While age and sex have often been considered, other key indicators may be neglected, which can lead to biased and ineffective models [27]. Several articles in this study noted limitations in being able to account for disease complexity and SV. Having a holistic understanding of the complexities in the system helps ensure essential components are considered. Causal loop diagrams can aid researchers in visualising priority relationships and feedback between variables in an entire system [50]. Nevertheless, data availability of key measures may be a limiting factor, as noted by several authors in this review. In the absence of data, proxy measures can be used if appropriate and results can be contextualised for the setting.

A limitation of our review is that it included articles written only in English. Furthermore, grey literature was not searched, nor were experts in the field consulted to identify additional publications. As aforementioned, the quality of the studies was not assessed given the focus on mapping the evidence. However, a form of quality control was to only include peer-reviewed publications. SV is a relatively new term from the disaster field in mostly HICs [7, 8]. While this may skew the article filtering to HICs, the database search included terms related to SV, such as health equity and SDH. While a standard definition and associated variables are still to be determined [8], there are many factors that contribute to SV. Articles that incorporated SV measures in their models but did not explicitly highlight them in their title or abstract were excluded during the screening process. Therefore, the results of this study may be underestimated. However, the aim of this study was to select articles that foreground SV, using the inclusion of key terms in the title and abstract as an indication of intention. In the absence of representative SV data, stratification may not be possible. One approach to include SV is to focus the whole model population on a specific vulnerable population and/or build the model at a level of granularity commensurate with representative data and then discuss the applicability to SV sub-populations. However, this was outside the scope, as this study aimed to understand how researchers incorporated SV into model methods and equations.

## Conclusions

The COVID-19 pandemic has caused a major setback to global poverty and social vulnerability, which is unprecedented in recent decades. The resulting crisis has been further worsened by climate shocks and conflicts among the world’s major food producers [9]. In the current context, incorporating social vulnerability in infectious disease mathematical modelling is of utmost importance. Models should consider social vulnerability to not only design more equitable interventions, but also to promote more effective infectious disease control and elimination strategies [27]. Select researchers have explored various methods to highlight social vulnerability in infectious disease modelling. While much attention has been paid to routine demographic variables and creating distinct models for different measures, there is potential to further the field. Our findings indicate social vulnerability is not considered holistically, often excluding important cultural and social behaviours that impact health outcomes. Transparency around data sources, consistency in reporting, collaboration with local experts, and studies focused on modelling cultural indicators are priorities for future research. The recognition of heterogeneities and inclusion of diverse measures of social vulnerability strengthens a mathematical model’s accuracy and utility and ensures models are more reflective of the world in which we live.

### Supplementary Information

**Additional file1.** Source data for analyses presented.

## Data Availability

All data generated or analysed during this study are included in this published article and its supplementary information files as Additional file 1.xls, which is the raw data collected during the full text data extraction.

## Appendix 1

### PubMed

("Communicable Diseases"[MeSH Terms] OR "infectious disease"[Text Word] OR "infectious diseases"[Text Word] OR "Communicable Diseases"[Text Word] OR "communicable disease"[Text Word] OR "outbreak"[Text Word] OR "outbreaks"[Text Word] OR "Pandemics"[MeSH Terms] OR "Pandemics"[Text Word] OR "pandemic"[Text Word] OR "Epidemics"[MeSH Terms] OR "Epidemics"[Text Word] OR "epidemic"[Text Word] OR "communicable diseases, emerging"[MeSH Terms] OR "emerging infectious diseases"[Text Word] OR "emerging communicable diseases"[Text Word] OR "emerging infectious disease"[Text Word] OR "re-emerging infectious diseases"[Text Word] OR "re-emerging infectious disease"[Text Word]).

AND

("models, theoretical"[MeSH Terms] OR "mathematical model"[Text Word] OR "mathematical models"[Text Word] OR "mathematical modeling"[Text Word] OR "mathematical modelling"[Text Word] OR "compartmental model"[Text Word] OR "compartmental models"[Text Word] OR "compartmental modeling"[Text Word] OR "compartmental modelling"[Text Word] OR "agent-based model"[Text Word] OR "agent-based models"[Text Word] OR "agent-based modeling"[Text Word] OR "agent-based modelling"[Text Word] OR "deterministic"[Text Word] OR "stochastic"[Text Word] OR "dynamic"[Text Word] OR "ordinary differential equation"[Text Word] OR "microsimulation"[Text Word] OR "infectious disease model"[Text Word] OR "infectious disease models"[Text Word] OR "infectious disease modeling"[Text Word] OR "infectious disease modelling"[Text Word] OR "SEIR"[Text Word] OR "SIR"[Text Word] OR "SIS"[Text Word] OR "SIRD"[Text Word] OR "SEIRD"[Text Word] OR "SIRV"[Text Word] OR "MSIR"[Text Word] OR "SEIS"[Text Word] OR "MSEIR"[Text Word]).

AND

("Social Vulnerability"[MeSH Terms] OR "Social Vulnerability"[Text Word] OR "Social Determinants of Health"[MeSH Terms] OR "Social Determinants of Health"[Text Word] OR "health equity"[Text Word] OR "health equality"[Text Word] OR "poverty"[Text Word] OR "socioeconomic"[Text Word] OR "socio-economic"[Text Word]).

### SCOPUS

TITLE-ABS-KEY ("infectious disease" OR "infectious diseases" OR "Communicable Diseases" OR "communicable disease" OR "outbreak" OR "outbreaks" OR "Pandemics" OR "pandemic" OR "Epidemics" OR "epidemic" OR "emerging infectious diseases" OR "emerging communicable diseases" OR "emerging infectious disease" OR "re-emerging infectious diseases" OR "re-emerging infectious disease").

AND

TITLE-ABS-KEY ("mathematical model" OR "mathematical models" OR "mathematical modeling" OR "mathematical modelling" OR "compartmental model" OR "compartmental models" OR "compartmental modeling" OR "compartmental modelling" OR "agent-based model" OR "agent-based models" OR "agent-based modeling" OR "agent-based modelling" OR "deterministic" OR "stochastic" OR "dynamic" OR "ordinary differential equation" OR "microsimulation" OR "infectious disease model" OR "infectious disease models" OR "infectious disease modeling" OR "infectious disease modelling" OR "SEIR" OR "SIR" OR "SIS" OR "SIRD" OR "SEIRD" OR "SIRV" OR "MSIR" OR "SEIS" OR "MSEIR").

AND

TITLE-ABS-KEY ("Social Vulnerability" OR "Social Determinants of Health" OR "health equity" OR "health equality" OR "poverty" OR "socioeconomic" OR "socio-economic").

### EBSCO Africa Wide Information

"infectious disease" OR "infectious diseases" OR "Communicable Diseases" OR "communicable disease" OR "outbreak" OR "outbreaks" OR "Pandemics" OR "pandemic" OR "Epidemics" OR "epidemic" OR "emerging infectious diseases" OR "emerging communicable diseases" OR "emerging infectious disease" OR "re-emerging infectious diseases" OR "re-emerging infectious disease"

AND

"mathematical model" OR "mathematical models" OR "mathematical modeling" OR "mathematical modelling" OR "compartmental model" OR "compartmental models" OR "compartmental modeling" OR "compartmental modelling" OR "agent-based model" OR "agent-based models" OR "agent-based modeling" OR "agent-based modelling" OR "deterministic" OR "stochastic" OR "dynamic" OR "ordinary differential equation" OR "microsimulation" OR "infectious disease model" OR "infectious disease models" OR "infectious disease modeling" OR "infectious disease modelling" OR "SEIR" OR "SIR" OR "SIS" OR "SIRD" OR "SEIRD" OR "SIRV" OR "MSIR" OR "SEIS" OR "MSEIR"

AND

"Social Vulnerability" OR "Social Determinants of Health" OR "health equity" OR "health equality" OR "poverty" OR "socioeconomic" OR "socio-economic"

### Cochrane Library

("infectious disease" OR "infectious diseases" OR "Communicable Diseases" OR "communicable disease" OR "outbreak" OR "outbreaks" OR "Pandemics" OR "pandemic" OR "Epidemics" OR "epidemic" OR "emerging infectious diseases" OR "emerging communicable diseases" OR "emerging infectious disease" OR "re-emerging infectious diseases" OR "re-emerging infectious disease") AND ("mathematical model" OR "mathematical models" OR "mathematical modeling" OR "mathematical modelling" OR "compartmental model" OR "compartmental models" OR "compartmental modeling" OR "compartmental modelling" OR "agent-based model" OR "agent-based models" OR "agent-based modeling" OR "agent-based modelling" OR "deterministic" OR "stochastic" OR "dynamic" OR "ordinary differential equation" OR "microsimulation" OR "infectious disease model" OR "infectious disease models" OR "infectious disease modeling" OR "infectious disease modelling" OR "SEIR" OR "SIR" OR "SIS" OR "SIRD" OR "SEIRD" OR "SIRV" OR "MSIR" OR "SEIS" OR "MSEIR") AND ("Social Vulnerability" OR "Social Determinants of Health" OR "health equity" OR "health equality" OR "poverty" OR "socioeconomic" OR "socio-economic").

### Clarivate Web of Science

TS = ("infectious disease" OR "infectious diseases" OR "Communicable Diseases" OR "communicable disease" OR "outbreak" OR "outbreaks" OR "Pandemics" OR "pandemic" OR "Epidemics" OR "epidemic" OR "emerging infectious diseases" OR "emerging communicable diseases" OR "emerging infectious disease" OR "re-emerging infectious diseases" OR "re-emerging infectious disease").

AND

TS = ("mathematical model" OR "mathematical models" OR "mathematical modeling" OR "mathematical modelling" OR "compartmental model" OR "compartmental models" OR "compartmental modeling" OR "compartmental modelling" OR "agent-based model" OR "agent-based models" OR "agent-based modeling" OR "agent-based modelling" OR "deterministic" OR "stochastic" OR "dynamic" OR "ordinary differential equation" OR "microsimulation" OR "infectious disease model" OR "infectious disease models" OR "infectious disease modeling" OR "infectious disease modelling" OR "SEIR" OR "SIR" OR "SIS" OR "SIRD" OR "SEIRD" OR "SIRV" OR "MSIR" OR "SEIS" OR "MSEIR").

AND

TS = ("Social Vulnerability" OR "Social Determinants of Health" OR "health equity" OR "health equality" OR "poverty" OR "socioeconomic" OR "socio-economic").

## Appendix 2

 Articles included in the final selection:

**Table:**

1	Adiga A, Chu S, Eubank S, et al. Disparities in spread and control of influenza in slums of Delhi: findings from an agent-based modelling study. BMJ Open. 2018;8(1):e017353. 10.1136/bmjopen-2017-017353.
2	Ajelli M, Merler S. An individual-based model of hepatitis A transmission. J Theor Biol. 2009;259(3):478–488. 10.1016/j.jtbi.2009.03.038.
3	Albi G, Pareschi L, Zanella M. Modelling lockdown measures in epidemic outbreaks using selective socio-economic containment with uncertainty. Math Biosci Eng. 2021;18(6):7161–7190. 10.3934/mbe.2021355.
4	Anderson SJ, Cherutich P, Kilonzo N, et al. Maximising the effect of combination HIV prevention through prioritisation of the people and places in greatest need: a modelling study. Lancet. 2014;384(9939):249–256. 10.1016/S0140-6736(14)61053-9.
5	Andersson KM, Owens DK, Vardas E, Gray GE, McIntyre JA, Paltiel AD. Predicting the impact of a partially effective HIV vaccine and subsequent risk behavior change on the heterosexual HIV epidemic in low- and middle-income countries: A South African example. J Acquir Immune Defic Syndr. 2007;46(1):78–90. 10.1097/QAI.0b013e31812506fd.
6	Araz OM, Galvani A, Meyers LA. Geographic prioritization of distributing pandemic influenza vaccines. Health Care Manag Sci. 2012;15(3):175–187. 10.1007/s10729-012-9199-6.
7	Ayoub HH, Chemaitelly H, Mumtaz GR, et al. Characterizing key attributes of COVID-19 transmission dynamics in China’s original outbreak: Model-based estimations. Glob Epidemiol. 2020;2:100,042. 10.1016/j.gloepi.2020.100042.
8	Banerjee S. Towards a Quantitative Model of Epidemics during Conflicts. Interdisciplinary Description of Complex Systems. 2019;17:598–614. 10.7906/indecs.17.3.16.
9	Bhunu CP, Mushayabasa S, Smith RJ. Assessing the effects of poverty in tuberculosis transmission dynamics. Appl Math Model. 2012;36(9):4173–4185. 10.1016/j.apm.2011.11.046.
10	Blake IM, Martin R, Goel A, et al. The role of older children and adults in wild poliovirus transmission. Proc Natl Acad Sci U S A. 2014;111(29):10604–10609. 10.1073/pnas.1323688111.
11	Bórquez A, Cori A, Pufall EL, et al. The Incidence Patterns Model to Estimate the Distribution of New HIV Infections in Sub-Saharan Africa: Development and Validation of a Mathematical Model. PLoS Medicine. 2016;13(9):e1002121. 10.1073/pnas.1323688111. 10.1371/journal.pmed.1002121.
12	Brand SPC, Ojal J, Aziza R, et al. COVID-19 transmission dynamics underlying epidemic waves in Kenya. Science. 2021;374(6570):989–994. 10.1126/science.abk0414.
13	Britton T, Ball F, Trapman P. A mathematical model reveals the influence of population heterogeneity on herd immunity to SARS-CoV-2. Science. 2020;369(6505):846–849. 10.1126/science.abc6810.
14	Brüningk SC, Klatt J, Stange M, et al. Determinants of SARS-CoV-2 transmission to guide vaccination strategy in an urban area. Virus Evol. 2022;8(1):veac002. 10.1093/ve/veac002.
15	Chang S, Pierson E, Koh PW, et al. Mobility network models of COVID-19 explain inequities and inform reopening. Nature. 2021;589(7840):82–87. 10.1038/s41586-020-2923-3.
16	Charpentier A, Elie R, Laurière M, Tran VC. COVID-19 pandemic control: balancing detection policy and lockdown intervention under ICU sustainability. Math Model Nat Phenom. 2020;15:57. 10.1051/mmnp/2020045.
17	Chen J, Chu S, Chungbaek Y, et al. Effect of modelling slum populations on influenza spread in Delhi. BMJ Open. 2016;6(9):e011699. 10.1136/bmjopen-2016-011699.
18	Chen SC, Chang CF, Jou LJ, Liao CM. Modelling vaccination programmes against measles in Taiwan. Epidemiol Infect. 2007;135(5):775–786. 10.1017/S0950268806007369.
19	Collins OC, Robertson SL, Govinder KS. Analysis of a waterborne disease model with socioeconomic classes. Math Biosci. 2015;269:86–93. 10.1016/j.mbs.2015.08.016.
20	Dangbé E, Irépran D, Perasso A, Békollé D. Mathematical modelling and numerical simulations of the influence of hygiene and seasons on the spread of cholera. Math Biosci. 2018;296:60–70. 10.1016/j.mbs.2017.12.004.
21	de Boer R, Lutscher F. Choice disability as a target for non-medical HIV intervention. Math Biosci. 2018;299:127–137. 10.1016/j.mbs.2018.03.015.
22	de Boer R, Lutscher F. The importance of choice disability and structural intervention in the HIV epidemic in Sub-Saharan Africa. PLoS One. 2017;12(4): e0175297. 10.1371/journal.pone.0175297.
23	de Oliveira RB, Rubio FA, Anderle R, et al. Incorporating social determinants of health into the mathematical modeling of HIV/AIDS. Sci Rep. 2022;12(1):20,541. 10.1038/s41598-022-24459-0.
24	Dimarco G, Pareschi L, Toscani G, Zanella M. Wealth distribution under the spread of infectious diseases. Phys Rev E. 2020;102(2):022303. 10.1103/PhysRevE.102.022303.
25	Dimka J, Sattenspiel L. “We didn’t get much schooling because we were fishing all the time”: Potential impacts of irregular school attendance on the spread of epidemics. Am J Hum Biol. 2021;34:e23578. 10.1002/ajhb.23578.
26	Dorratoltaj N, Marathe A, Lewis BL, Swarup S, Eubank SG, Abbas KM. Epidemiological and economic impact of pandemic influenza in Chicago: Priorities for vaccine interventions. PLoS Comput Biol. 2017;13(6):e1005521. 10.1371/journal.pcbi.1005521.
27	Dover DC, Kirwin EM, Hernandez-Ceron N, Nelson KA. Pandemic Risk Assessment Model (PRAM): a mathematical modeling approach to pandemic influenza planning. Epidemiol Infect. 2016;144(16):3400–3411. 10.1017/S095026881600185.
28	Durante R, Guiso L, Gulino G. Asocial capital: Civic culture and social distancing during COVID-19. J Public Econ. 2021;194:104,342. 10.1016/j.jpubeco.2020.104342.
29	Dutta R, Gomes SN, Kalise D, Pacchiardi L. Using mobility data in the design of optimal lockdown strategies for the COVID-19 pandemic. PLoS Comput Biol. 2021;17(8):e1009236. 10.1371/journal.pcbi.1009236.
30	Enns EA, Brandeau ML. Inferring model parameters in network-based disease simulation. Health Care Manag Sci. 2011;14(2):174–188. 10.1007/s10729-011-9150-2.
31	Epstein JM, Parker J, Cummings D, Hammond RA. Coupled Contagion Dynamics of Fear and Disease: Mathematical and Computational Explorations. PLoS One. 2008;3(12):e3955. 10.1371/journal.pone.0003955.
32	Esra RT, Johnson LF. Modelling the impact of screening for chlamydia and gonorrhoea in youth and other high-prevalence groups in a resource-limited setting. Int J Public Health. 2020;65(4):413–423. 10.1007/s00038-020-01351-0.
33	Fazio M, Pluchino A, Inturri G, Le Pira M, Giuffrida N, Ignaccolo M. Exploring the impact of mobility restrictions on the COVID-19 spreading through an agent-based approach. J Transp Health. 2022;25:101,373. 10.1016/j.jth.2022.101373.
34	Galanis G, Hanieh A. Incorporating Social Determinants of Health into Modelling of COVID-19 and other Infectious Diseases: A Baseline Socio-economic Compartmental Model. Soc Sci Med. 2021;274:113,794. 10.1016/j.socscimed.2021.113794.
35	Goedel WC, Bessey S, Lurie MN, et al. Projecting the impact of equity-based preexposure prophylaxis implementation on racial disparities in HIV incidence among MSM. AIDS. 2020;34(10):1509–1517. 10.1097/QAD.0000000000002577.
36	Gozzi N, Bajardi P, Perra N. The importance of non-pharmaceutical interventions during the COVID-19 vaccine rollout. PLoS Comput Biol. 2021;17(9):e1009346. 10.1371/journal.pcbi.1009346.
37	Grefenstette JJ, Brown ST, Rosenfeld R, et al. FRED (A Framework for Reconstructing Epidemic Dynamics): an open-source software system for modeling infectious diseases and control strategies using census-based populations. BMC Public Health. 2013;13(1):940. 10.1186/1471-2458-13-940.
38	Hallett TB, Gregson S, Mugurungi O, Gonese E, Garnett GP. Assessing evidence for behaviour change affecting the course of HIV epidemics: A new mathematical modelling approach and application to data from Zimbabwe. Epidemics. 2009;1(2):108–117. 10.1016/j.epidem.2009.03.001.
39	Hallett TB, Singh K, Smith JA, White RG, Abu-Raddad LJ, Garnett GP. Understanding the Impact of Male Circumcision Interventions on the Spread of HIV in Southern Africa. PLoS One. 2008;3(5):e2212. 10.1371/journal.pone.0002212.
40	He D, Wang X, Gao D, Wang J. Modeling the 2016–2017 Yemen cholera outbreak with the impact of limited medical resources. Journal of Theoretical Biology. 2018;451:80–85. 10.1016/j.jtbi.2018.04.041.
41	Hou X, Gao S, Li Q, et al. Intracounty modeling of COVID-19 infection with human mobility: Assessing spatial heterogeneity with business traffic, age, and race. Proc Natl Acad Sci U S A. 2021;118(24):e2020524118. 10.1073/pnas.2020524118.
42	Hu H, Gong P, Xu B. Spatially explicit agent-based modelling for schistosomiasis transmission: human–environment interaction simulation and control strategy assessment. Epidemics. 2010;2(2):49–65. 10.1016/j.epidem.2010.03.004.
43	Huang X, Clements ACA, Williams G, Mengersen K, Tong S, Hu W. Bayesian estimation of the dynamics of pandemic (H1N1) 2009 influenza transmission in Queensland: A space–time SIR-based model. Environ Res. 2016;146:308–314. 10.1016/j.envres.2016.01.013.
44	Hunter E, Mac Namee B, Kelleher JD. Using a Socioeconomic Segregation Burn-in Model to Initialise an Agent-Based Model for Infectious Diseases. JASSS. 2018;21(4):9. 10.18564/jasss.3870
45	Hyder A, Leung B. Social deprivation and burden of influenza: Testing hypotheses and gaining insights from a simulation model for the spread of influenza. Epidemics. 2015;11:71–79. 10.1016/j.epidem.2015.03.004.
46	Joshi H, Lenhart S, Albright K, Gipson K. Modeling the effect of information campaigns on the HIV epidemic in Uganda. Math Biosci Eng. 2008;5(4):757–770. 10.3934/mbe.2008.5.757.
47	Kerr CC, Stuart RM, Gray RT, et al. Optima: A Model for HIV Epidemic Analysis, Program Prioritization, and Resource Optimization. J Acquir Immune Defic Syndr. 2015;69(3):365–376. 10.1097/QAI.0000000000000605.
48	Khadadah F, Al-Shammari AA, Alhashemi A, et al. The effects of non-pharmaceutical interventions on SARS-CoV-2 transmission in different socioeconomic populations in Kuwait: a modeling study. BMC Public Health. 2021;21(1):990. 10.1186/s12889-021-10984-6.
49	Kombe IK, Munywoki PK, Baguelin M, Nokes DJ, Medley GF. Model-based estimates of transmission of respiratory syncytial virus within households. Epidemics. 2019;27:1–11. 10.1016/j.epidem.2018.12.001.
50	Kumar S, Piper K, Galloway DD, Hadler JL, Grefenstette JJ. Is population structure sufficient to generate area-level inequalities in influenza rates? An examination using agent-based models. BMC Public Health. 2015;15(1):947. 10.1186/s12889-015-2284-2.
51	Lawson AB, Kim J. Space–time covid-19 Bayesian SIR modeling in South Carolina. PLoS One. 2021;16(3):e0242777. 10.1371/journal.pone.0242777.
52	Liu F, Enanoria WTA, Zipprich J, et al. The role of vaccination coverage, individual behaviors, and the public health response in the control of measles epidemics: an agent-based simulation for California. BMC Public Health. 2015;15(1):447. 10.1186/s12889-015-1766-6.
53	Liu R, Wu J, Zhu H. Media/Psychological Impact on Multiple Outbreaks of Emerging Infectious Diseases. Comput Math Methods Med. 2007;8:153–164. 10.1080/17486700701425870.
54	López L, Burguerner G, Giovanini L. Addressing population heterogeneity and distribution in epidemics models using a cellular automata approach. BMC Research Notes. 2014;7(1):234. 10.1186/1756-0500-7-234.
55	Medlock J, Galvani AP. Optimizing influenza vaccine distribution. Science. 2009;325(5948):1705–1708. 10.1126/science.1175570.
56	Menkir TF, Jbaily A, Verguet S. Incorporating equity in infectious disease modeling: Case study of a distributional impact framework for measles transmission. Vaccine. 2021;39(21):2894–2900. 10.1016/j.vaccine.2021.03.023.
57	Mistry D, Litvinova M, Pastore y Piontti A, et al. Inferring high-resolution human mixing patterns for disease modeling. Nat Commun. 2021;12(1):323. 10.1038/s41467-020-20544-y.
58	Mubayi A, Paredes M, Ospina J. A Comparative Assessment of Epidemiologically Different Cutaneous Leishmaniasis Outbreaks in Madrid, Spain and Tolima, Colombia: An Estimation of the Reproduction Number via a Mathematical Model. Trop Med Infect Dis. 2018;3:43. 10.3390/tropicalmed3020043
59	Munday JD, van Hoek AJ, Edmunds WJ, Atkins KE. Quantifying the impact of social groups and vaccination on inequalities in infectious diseases using a mathematical model. BMC Med. 2018;16(1):162. 10.1186/s12916-018-1152-1
60	Musa SS, Qureshi S, Zhao S, Yusuf A, Mustapha UT, He D. Mathematical modeling of COVID-19 epidemic with effect of awareness programs. Infect Dis Model. 2021;6:448–460. 10.1016/j.idm.2021.01.012.
61	Mushayabasa S, Bhunu C, Schwartz E, Magombedze G, Tchuenche M. Socio-economic status and HIV/AIDS dynamics: A modeling approach. World J Model Simul. 2011;7:243–257
62	Mutua JM, Barker CT, Vaidya NK. Modeling Impacts of Socioeconomic Status and Vaccination Programs on Typhoid Fever Epidemics. Electron J Differ Eq. 2017:63–74
63	Myers K, Redere A, Fefferman NH. How resource limitations and household economics may compromise efforts to safeguard children during outbreaks. BMC Public Health. 2020;20(1):270. 10.1186/s12889-019-7968-6.
64	Patterson-Lomba O, Safan M, Towers S, Taylor J. Modeling the role of healthcare access inequalities in epidemic outcomes. Math Biosci Eng. 2016;13(5):1011–1041. 10.3934/mbe.2016028.
65	Pedro SA, Tchuenche JM. HIV/AIDS dynamics: impact of economic classes with transmission from poor clinical settings. J Theor Biol. 2010;267(4):471–485. 10.1016/j.jtbi.2010.09.019.
66	Poletti P, Merler S, Ajelli M, et al. Evaluating vaccination strategies for reducing infant respiratory syncytial virus infection in low-income settings. BMC Med. 2015;13(1):49. 10.1186/s12916-015-0283-x.
67	Prem K, Liu Y, Russell TW, et al. The effect of control strategies to reduce social mixing on outcomes of the COVID-19 epidemic in Wuhan, China: a modelling study. Lancet Public Health. 2020;5(5):e261-e270. 10.1016/S2468-2667(20)30073-6.
68	Rehkopf D, Furumoto-Dawson A, Kiszewski A, Awerbuch-Friedlander T. Spatial Spread of Tuberculosis through Neighborhoods Segregated by Socioeconomic Position: A Stochastic Automata Model. Discrete Dyn Nat Soc. 2015;2015:e583819. 10.1155/2015/583819.
69	Robinson B, Edwards J, Kendzerska T, et al. Comprehensive compartmental model and calibration algorithm for the study of clinical implications of the population-level spread of COVID-19: a study protocol. BMJ Open. 2022;12:e052681. 10.1136/bmjopen-2021-05268.
70	Romaszko J, Siemaszko A, Bodzioch M, Buciński A, Doboszyńska A. Active Case Finding Among Homeless People as a Means of Reducing the Incidence of Pulmonary Tuberculosis in General Population. Adv Exp Med Biol. 2016;911:67–76. 10.1007/5584_2016_225.
71	Sahasranaman A, Jensen HJ. Poverty in the time of epidemic: A modelling perspective. PLoS One. 2020;15(11):e0242042. 10.1371/journal.pone.0242042.
72	Sahasranaman A, Jensen HJ. Spread of COVID-19 in urban neighbourhoods and slums of the developing world. J R Soc Interface. 2021;18(174):20200599. 10.1098/rsif.2020.0599.
73	Sato AH, Ito I, Sawai H, Iwata K. An epidemic simulation with a delayed stochastic SIR model based on international socioeconomic-technological databases. 2015:2732–2741. 10.1109/BigData.2015.7364074.
74	Shrestha S, Chihota V, White RG, Grant AD, Churchyard GJ, Dowdy DW. Impact of Targeted Tuberculosis Vaccination Among a Mining Population in South Africa: A Model-Based Study. Am J Epidemiol. 2017;186(12):1362–1369. 10.1093/aje/kwx192.
75	Skrip LA, Fallah MP, Bedson J, Hébert-Dufresne L, Althouse BM. Coordinated support for local action: Modeling strategies to facilitate behavior adoption in urban-poor communities of Liberia for sustained COVID-19 suppression. Epidemics. 2021;37:100,529. 10.1016/j.epidem.2021.100529.
76	Springborn M, Chowell G, MacLachlan M, Fenichel EP. Accounting for behavioral responses during a flu epidemic using home television viewing. BMC Infectious Diseases. 2015;15(1):21. 10.1186/s12879-014-0691-0.
77	Stover J, Glaubius R, Teng Y, et al. Modeling the epidemiological impact of the UNAIDS 2025 targets to end AIDS as a public health threat by 2030. PLoS Medicine. 2021;18(10):e1003831. 10.1371/journal.pmed.1003831.
78	Stroud P, Del Valle S, Sydoriak S, Riese J, Mniszewski S. Spatial Dynamics of Pandemic Influenza in a Massive Artificial Society. J Artif Soc Soc Simul. 2007;10
79	Timpka T, Morin M, Jenvald J, Gursky E, Eriksson H. Dealing with ecological fallacy in preparations for influenza pandemics: use of a flexible environment for adaptation of simulations to household structures in local contexts. Stud Health Technol Inform. 2007;129(Pt 1):218–222
80	Torres-Sorando L, Rodríguez DJ. Models of spatio-temporal dynamics in malaria. Ecol Modell. 1997;104(2):231–240. 10.1016/S0304-3800(97)00135-X.
81	Tuite AR, Fisman DN, Kwong JC, Greer AL. Optimal Pandemic Influenza Vaccine Allocation Strategies for the Canadian Population. PLoS One. 2010;5(5):e10520. 10.1371/journal.pone.0010520.
82	van Zandvoort K, Jarvis CI, Pearson CAB, et al. Response strategies for COVID-19 epidemics in African settings: a mathematical modelling study. BMC Med. 2020;18(1):324. 10.1186/s12916-020-01789-2.
83	Wallace R. A fractal model of HIV transmission on complex socio-geographic networks. Part 2: spread from a ghettoized “core group” into a “general population.” Environ Plan A. 1994;26(5):767–778. 10.1068/a260767.
84	Wanduku D. A nonlinear multi-population behavioral model to assess the roles of education campaigns, random supply of aids, and delayed ART treatment in HIV/AIDS epidemics. Math Biosci Eng. 2020;17(6):6791–6837. 10.3934/mbe.2020354.
85	Wang L, Chen J, Marathe A. A framework for discovering health disparities among cohorts in an influenza epidemic. World Wide Web. 2019;22(6):2997–3020. 10.1007/s11280-018-0608-8
86	Yang HM, Ferreira MU. Assessing the effects of global warming and local social and economic conditions on the malaria transmission. Rev Saude Publica. 2000;34(3):214–222. 10.1590/s0034-89102000000300002.
87	Yechezkel M, Weiss A, Rejwan I, Shahmoon E, Ben-Gal S, Yamin D. Human mobility and poverty as key drivers of COVID-19 transmission and control. BMC Public Health. 2021;21(1):596. 10.1186/s12889-021-10561-x.
88	Yu Z, Liu J, Wang X, Zhu X, Wang D, Han G. Efficient Vaccine Distribution Based on a Hybrid Compartmental Model. PLoS One. 2016;11(5):e0155416. 10.1371/journal.pone.0155416.
89	Zhang WB, Ge Y, Liu M, et al. Risk assessment of the step-by-step return-to-work policy in Beijing following the COVID-19 epidemic peak. Stoch Environ Res Risk Assess. 2021;35(2):481–498. 10.1007/s00477-020-01929-3.

## Abbreviations

ABM: Agent-based model
CDC: Centers for Disease Control and Prevention
HIC: High-income country
KAP: Knowledge, attitudes, and practices
LMIC: Low- and middle-income country
PRISMA-ScR: Preferred Reporting Items for Systematic reviews and Meta-Analyses extension for Scoping Reviews
SDH: Social determinants of health
STI: Sexually transmitted infection
SV: Social vulnerability
UK: United Kingdom
US: United States of America
